# Craniofacial Fibrous Dysplasia of Zygomaticomaxillary Complex

**Published:** 2017-09

**Authors:** Kumar Nilesh, Prashant Punde, MI Parkar

**Affiliations:** Department of Oral and Maxillofacial Surgery, School of Dental Sciences, Krishna Institute of Medical Sciences University, Krishna Hospital, Maharashtra, India

**Keywords:** Maxillary bone disorder, Maxillary sinus, Fibro-osseous disease

## Abstract

Fibrous dysplasia is a benign bone disease first described by Lichtenstein in 1938. It is characterized by progressive replacement of normal bone with fibro-osseous connective tissue. When the disease involves craniofacial skeleton, it results in significant disfigurement and other functional problems. This paper reports a case of large craniofacial fibrous dysplasia involving zygomaticomaxillary complex in a 24-year old male patient. Clinical presentation and imaging characteristics of the pathology is discussed in detail. The disease caused significant facial asymmetry which was satisfactorily managed by surgical recontouring.

## INTRODUCTION

Fibrous dysplasia (FD) is a non-malignant disorder of bone caused by somatic mutations of gene which encodes for the alpha subunit of G protein.^1^ The genetic mutation results in metabolic disorder characterized by replacement of normal bone marrow by fibrous tissue and haphazardly distributed woven bone. FD is a rare disorder with reported incidence varying from 1:4000 to 1:10,000. It accounts for approximately 2.5% of all bone lesions and about 7% of all benign bone tumors.^2^

Depending on the number of bones involved, FD is divided into two subtypes- monostotic fibrous dysplasia (MFD) and polyostotic fibrous dysplasia (PFD). As the names indicate MFD is characterized by involvement of a single bone, whereas PFD involves two or more bones. Polyostotic form commonly involves craniofacial bones, proximal femur, and rib. About 3% of PFD is associated with endocrinopathies and skin pigmentation (café au lait spots) and is termed as McCune-Albright syndrome. MFD is most common form and accounts for 80 to 85% cases.^3^

FD affecting the craniofacial skeleton although may present as a single lesion, cannot be truly categorized as monostotic because the pathology is not limited to a single bone, and often involve two or more adjacent bones. The term “craniofacial fibrous dysplasia” (CFD) is used to describe such lesions which are confined to contiguous bones of the craniofacial skeleton.^3^ This paper reports a case of large CFD involving zygomaticomaxillary complex. The clinical presentation, imaging characteristics and management of the pathology are described. 

## CASE REPORT

A biopsy proven case of fibrous dysplasia of facial skeleton, in a 24 years old man was referred to Oral and Maxillofacial Surgery Clinic, Krishna Hospital, Karad for further evaluation and management. The patient presented with a chief complaint of a painless swelling over left side of face. He first noticed the swelling about twelve years back and it had gradually increased to the present size. The swelling had reportedly not increased in size since last two years. Patient’s medical history was non-contributory. There was no previous history of trauma. 

**Fig. 1:**
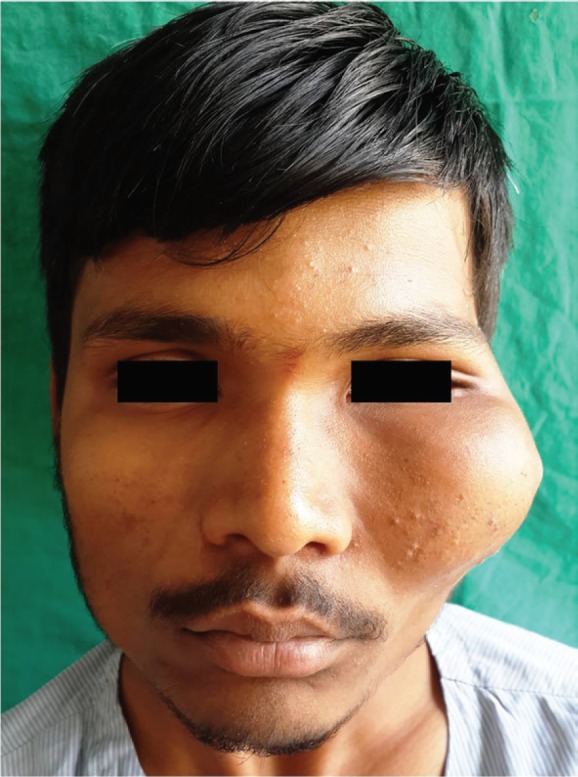
Clinical presentation of the lesion

On examination, a non-tender, bony hard swelling was noted, involving almost entire left mid-face, extending from midline to the lateral canthus of eye mediolaterally and from infra-orbital rim to the level of upper lip, measuring about 7 cm in diameter (Figure 1). The skin over the swelling appeared stretched and the lateral canthus of left eye was laterally displaced. Ophthalmic evaluation revealed no abnormality and visual acuity was normal. No similar lesion was detected elsewhere in the body. Systemic evaluation of the patient revealed no systemic disorders or endocrinopathy. 

**Fig. 2:**
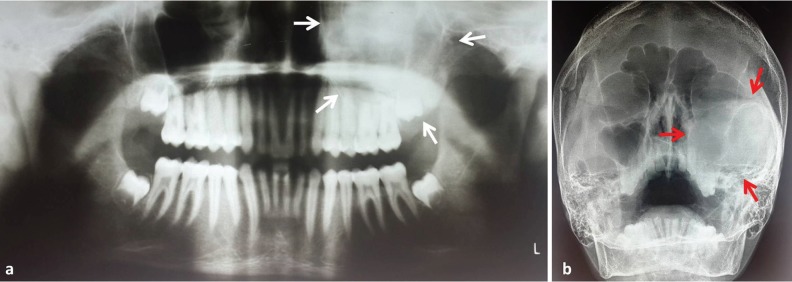
Orthopantomogram (a) and paranasal sinus view (b) showing homogenous radiopaque lesion of left maxilla.

Orthopantomogram (OPG) showed a homogenous radiopaque lesion involving left maxilla. The trabacular pattern was altered, with numerous, irregular, short and immature appearing trabeculae, giving an opacified *ground glass* appearance. Surrounding area was normal with no evidence of radiolucent capsule (Figure 2a). Paranasal sinus view (PNS) showed well-defined homogenous radiopaque lesion involving entire left maxillary sinus, measuring approximately 5×6 cm in size, extending superiorly till floor of the orbital fossa. The posterior extent of lesion was not clearly appreciated in this view. No radiolucent areas were seen within the radiopacity and the surrounding areas appeared normal (Figure 2b).

**Fig. 3:**
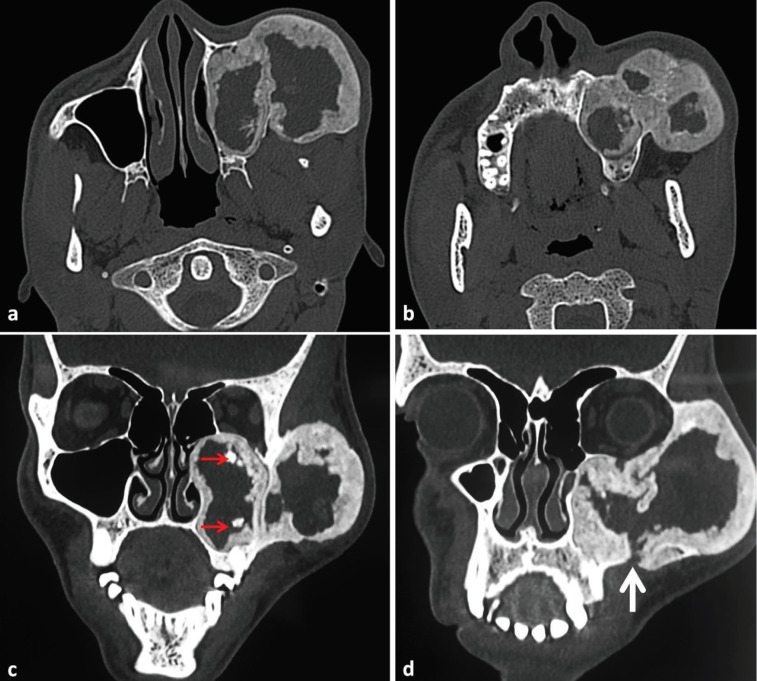
Axial (a, b) and coronal (c, d) sections of computed tomography scan showing well-defined multilobulated expansile lesion involving the left zygoma, lateral orbital rim, infra-orbital rim, zygomatic arch, maxillary sinus and superior alveolar ridge. Foci of ossification noted within the lesion (red arrow) along with area of breach of bone at zygomatic buttress area (white arrow) suggestive of previous site of biopsy

Computed tomography (CT) scan showed well-defined multilobulated expansile lesion involving the left zygoma, lateral orbital rim (zygomatic process of frontal bone and frontal process of zygomatic bone), infra-orbital rim, zygomatic arch, maxillary sinus and superior alveolar ridge, measuring about 50×30×45 mm. Internal structure of the pathology (in axial and coronal sections) showed peripheral ground glass appearance, surrounding a central zone of fibrous (woven) bone. Areas of ossification were noted within the lesion (Figure 3). 

**Fig. 4:**
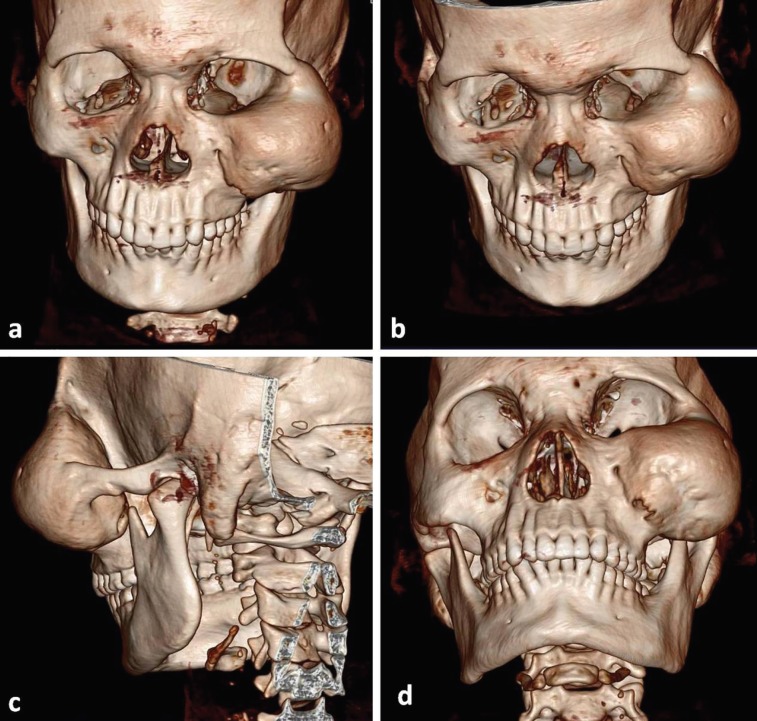
3D reconstruction images showing a large bony lesion involving left ZMC

**Fig. 5:**
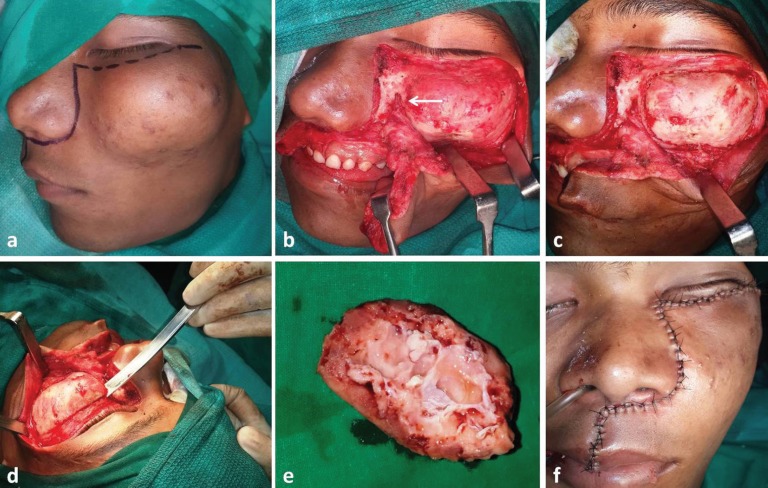
Intra-operative photographs showing modified Weber Ferguson incision (a) to approach the lesion, exposure of the underlying bony mass (b), osteotomy marked and bone recontoured (c, d), excised bone (e) and closure of the site (f).

Three dimensional reconstructions of CT scan images showed large bony lesion of left ZMC, producing a contour bulge over maxilla, lateral orbital rim, orbital floor and zygoma (Figure 4). The clinical and radiological presentation of the pathology was suggestive of CFD involving zygomaticomaxillary complex. Surgical recontouring of the lesion was planned under general anesthesia. Weber Ferguson incision with lateral canthus extension was used to approach the lesion. The lesion was recountoured taking care to identify and protect the infra-orbital nerve and contents of orbit (Figure 5). 

**Fig. 6:**
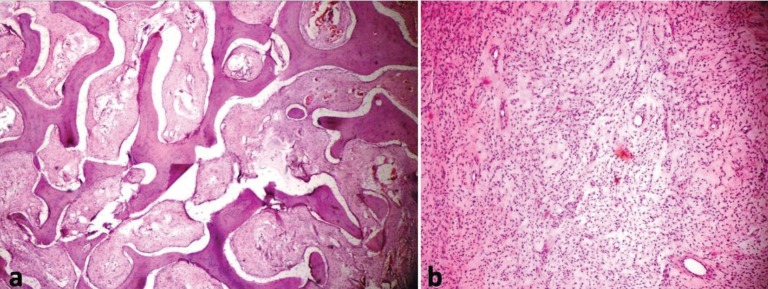
Photomicrograph showing (a) Chinese letter shaped trabeculae in a highly cellular connective tissue stroma (H&E, 10×), (b) Well vascularized stroma with dilated vascular channels (H&E, 40×).

**Fig. 7:**
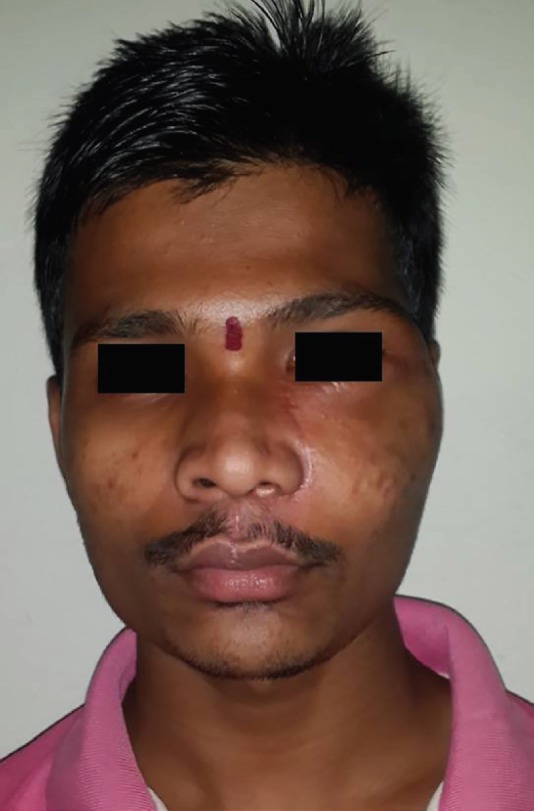
Two years postoperative clinical photograph showing the reestablished facial symmetry

After adequate reduction of the mass, to attain best possible facial symmetry, the flap was closed in layers. The patient had uneventful postoperative recovery. Histopathological examination of the excised bone specimen showed loose cellular fibrous stroma composed of haphazardly arranged trabeculae of woven bone giving the characteristic Chinese letter pattern, confirming the diagnosis of fibrous dysplasia (Figure 6). The patient was followed up at regular intervals and showed acceptable facial symmetry with no evidence of increase in size of the pathology or recurrence at 2-year postoperative period (Figure 7). 

## DISCUSSION

Fibrous dysplasia is a rare benign bone disease. When it involves the craniofacial skeleton it causes significant morphological, functional and aesthetic disturbances. Craniofacial fibrous dysplasia (CFD) most commonly involves zygomaticomaxillary complex as seen in present case. The other bones commonly involved include cranial base, maxilla and mandible.^4^ Signs and symptoms of CFD vary depending on the location and growth of the lesion. The natural progression of the disease is usually slow indolent mass lesion. Slow growing early lesion may get diagnosed during routine clinical examination or as an incidental finding on head and neck radiographs. 

Although rare, rapid growth with cortical bone expansion and displacement of adjacent vital structures maybe seen in young children and pre-pubertal adolescents. Progression of the lesions invariably taper off as the patients approach puberty. Although continued active disease is uncommon in adulthood, they have been reported.^5^ The present case produced a large painless bony mass over mid-face causing significant facial asymmetry and disfigurement. Visual disturbances, hearing impairment, nasal obstruction, pain, paresthesia, and malocclusion are other possible presenting symptoms of CFD though none of these were noted in our case. The only complaint our patient had was of esthetic disfigurement due to the facial asymmetry.

Radiographically the appearance of FD varies from a diffuse radiopaque mass, or a mixed lesion or a radiolucency of the involved bone(s), depending upon the stage and progression of the disease. Sontake et al in a review of fibrous dysplasia involving maxillofacial skeleton stated that ground glass appearance was the most common radiographic presentation of internal structure of FD. CFD involving the maxillary sinus may cause cortical expansion over the external surface. Internal expansion of the lesion within the maxillary sinus leads to a reduction in the volume of sinus cavity, although the shape usually remains unaltered.^6^

In the reported case the lesion presented as uniform well-defined homogenous radiopaque mass on plain radiography, giving typical ground glass appearance. Internal structure of the pathology on computed tomography showed peripheral ground glass appearance, surrounding a central zone of fibrous (woven) bone with areas of hyperdensity (ossification). History, clinical presentation and the radiographic features are often adequate to establish diagnosis of FD. 

Histologically FD shows low to moderately cellular fibrous stroma surrounding irregular, curvilinear trabeculae of woven bone, which is arranged in a pattern commonly referred to as Chinese letter form. The stroma of the lesion has varying amount of collagen, with ratio of fibrous tissue to bone ranging from being totally fibrous to being densely packed with dysplastic trabeculae. The microscopic features cannot differentiate various forms of FD and does not predict the biological behavior of these lesions.^7^ The primary goal in management of patients with CFD is to correct the bone deformity so as to restore the facial contour and symmetry. Surgical management of the lesion varies from conservative shaving or osseous contouring to more aggressive resection of the lesion in-toto. 

It is recommended that all the contouring procedure should be carried out after end of the growth phase (i.e. after adolescence). Patients should be informed about the risk of recurrence and need for surgical recontouring or resection in case of growth of the lesion. Satisfactory bone shaping of the lesion was attained for the present case using extended Weber Ferguson approach. Patient at two years follow-up showed no increase in size of the lesion with aesthetically improved facial symmetry and appearance and was very satisfied with his appearance. Thus this paper highlights the features, both clinical and imaging based, to be borne in mind when handling such patients with facial asymmetry. It highlights the role of surgical options that can be made use of to deal with fibrous dysplasia.

## CONFLICT OF INTEREST

The authors declare no conflict of interest.
